# Public Response to a Social Media Tobacco Prevention Campaign: Content Analysis

**DOI:** 10.2196/20649

**Published:** 2020-12-07

**Authors:** Anuja Majmundar, NamQuyen Le, Meghan Bridgid Moran, Jennifer B Unger, Katja Reuter

**Affiliations:** 1 Department of Preventive Medicine Keck School of Medicine University of Southern California Los Angeles, CA United States; 2 Southern California Clinical and Translational Science Institute Keck School of Medicine University of Southern California Los Angeles, CA United States; 3 Department of Health, Behavior & Society Johns Hopkins University Bloomberg School of Public Health Balltimore, MD United States

**Keywords:** social media, health campaign, tobacco, online, health communication, internet, Twitter, Facebook, Instagram

## Abstract

**Background:**

Prior research suggests that social media–based public health campaigns are often targeted by countercampaigns.

**Objective:**

Using reactance theory as the theoretical framework, this research characterizes the nature of public response to tobacco prevention messages disseminated via a social media–based campaign. We also examine whether agreement with the prevention messages is associated with comment tone and nature of the contribution to the overall discussion.

**Methods:**

User comments to tobacco prevention messages, posted between April 19, 2017 and July 12, 2017, were extracted from Twitter, Facebook, and Instagram. Two coders categorized comments in terms of tone, agreement with message, nature of contribution, mentions of government agency and regulation, promotional or spam comments, and format of comment. Chi-square analyses tested associations between agreement with the message and tone of the public response and the nature of contributions to the discussions.

**Results:**

Of the 1242 comments received (Twitter: n=1004; Facebook: n=176; Instagram: n=62), many comments used a negative tone (42.75%) and disagreed with the health messages (39.77%), while the majority made healthy contributions to the discussions (84.38%). Only 0.56% of messages mentioned government agencies, and only 0.48% of the comments were antiregulation. Comments employing a positive tone (84.13%) or making healthy contributions (69.11%) were more likely to agree with the campaign messages (*P*=0.01). Comments employing a negative tone (71.25%) or making toxic contributions (36.26%) generally disagreed with the messages (*P*=0.01).

**Conclusions:**

The majority of user comments in response to a tobacco prevention campaign made healthy contributions. Our findings encourage the use of social media to promote dialogue about controversial health topics such as smoking. However, toxicity was characteristic of comments that disagreed with the health messages. Managing negative and toxic comments on social media is a crucial issue for social media–based tobacco prevention campaigns to consider.

## Introduction

### Overview

Protobacco messages outnumber antitobacco messages on social media, which raises concerns about their effects on vulnerable populations such as youth [1-3]. Adolescents who engage with tobacco-related content online are more likely to initiate tobacco use and less likely to support tobacco-related regulations [4-6]. Social media accounts associated with tobacco companies, influencers, tobacco enthusiasts, and automated bots (algorithms that automatically produce content and engage with legitimate human accounts on social media) create and disseminate protobacco information online [7-10]. Although sites such as Facebook, Twitter, Instagram, and Google prohibit tobacco marketing, social media users can still view protobacco information in the form of news articles, discussion forums, posts from tobacco retailers, and brand (paid) and organic (unpaid) posts about tobacco use from peers.

Evidence-based tobacco prevention campaigns could play a crucial role in countering the effects and volume of protobacco messages at scale. However, negative public response to such messages from protobacco individuals posting negative comments can potentially undermine these efforts. Real-time surveillance of online health communication campaigns (eg, analysis of metadata such as likes and shares or qualitative data such as comments and posts), although limited, has highlighted negative public responses to tobacco prevention messages. Recent evidence suggests that public responses and organized protobacco groups create a large volume of organic (unpaid) social media messages that are against tobacco awareness campaigns [11,12]. Allem et al [11] found that a countercampaign to California’s “Still Blowing Smoke” campaign, “Not Blowing Smoke,” questioned health claims and raised objections to electronic cigarette regulations. In another instance, Chicago’s tobacco policy campaign was countered using an “astroturfing” strategy, wherein large numbers of bot-generated countermessages conveyed a false consensus that the public disagreed with the policy [12]. Although such a response to health messages on social media is concerning, it also points to missed opportunities of social media engagement directed towards creating dialogue with vulnerable audiences. Social media yields high reach and elicits engagement and activism from audiences [13,14]. To leverage these opportunities, it is crucial to characterize the nature of public responses using a broader sample of tobacco prevention messages and to devise strategies to address negative comments in the future.

### Theoretical Framework

The reactance theory provides a useful framework to explain negative public responses to tobacco prevention messages [15-19]. According to reactance theory, when individuals encounter messages that they perceive to threaten their freedom of choice, they experience a motivational state of reactance and act in ways to recover or assert their lost or threatened freedom [20]. Exposure to antitobacco message features may threaten individuals’ perceived freedom to smoke and thereby introduce psychological reactance, which results in negative responses and resistance to such messages. Research also suggests that threat to freedom enhances the attractiveness of the threatened freedom (eg, smoking) and thereby results in higher intentions to exercise that freedom [21]. Tobacco prevention messages, in this context, can potentially threaten multiple free behaviors, such as freedom to smoke and to self-identity as a smoker, and can potentially increase the likelihood of performing unhealthy behaviors [20].

Resistance to tobacco prevention messages may polarize debates on social media [22]. Past evidence suggests that polarized public discussions on social media are marked with one-sided perspectives related to health topics. For instance, Allem et al [11] demonstrated that groups of Twitter users emphasized the potential benefits of electronic cigarettes for cessation but not the potential risks.

### Social Media–Based Health Communication

Each social media platform offers unique features for health communication initiatives. As noted in previous research, Facebook can elicit interaction with campaign followers, engagement with health facts and health myths, and the possibility of creating a closed-group communication in the case of sensitive health-related topics such as HIV [23]. Twitter offers instant diffusion of health messages that may lose links with the sources when Twitter users share health messages with their network members, and those network members consequently share the messages with their own networks [24]. Studies evaluating campaigns on Instagram, although limited, suggest that posts with embedded health messages are linked to high perceived message effectiveness [25].

Given the importance of social media campaigns for tobacco education and prevention and the potential for user-generated comments to undermine these efforts, it is critical to understand the nature of such comments. To address this need, this study undertook a content analysis of public response to a semiautomated tobacco prevention campaign on Twitter, Facebook, and Instagram, which was described in detail in a technical paper by Reuter et al [15]. We characterized the nature of these comments along the following 7 dimensions: (1) tone of the comment, (2) nature of contribution, (3) agreement with the prevention message, (4) mentions of government agency, (5) policy/regulation, (6) promotion/spam, and (7) format of comment. We hypothesized that agreement with the prevention messages is significantly associated with the tone of the comment and nature of the contribution.

## Methods

### Overview of the Campaign

The campaign was live on 3 social media platforms (Twitter, Facebook, and Instagram) from April 19, 2017 to July 12, 2017. Campaign messages comprised of 102 parametrized message templates (defined as messages that fit within the limitations and parameters [eg, character count] of each social media platform) from two government-sponsored health education campaigns on the risks of combustible tobacco products. See Multimedia Appendix 1 for the complete list of parameterized message templates used in this study. We randomized the message templates within a pool of 226 unique images sourced from government-sponsored campaigns or, in the case of copyright protected campaign images, representative stock images from an online platform, Stocksnap.

A total of 1275 campaign message posts were posted during the study period (Twitter: n=510; Facebook: n=510; Instagram: n=255) as specified in the technical implementation paper published previously [26]. Each campaign message was posted at the most once each month over 85 days. We used a web-based tool (Trial Promoter) developed previously by our team of researchers to disseminate randomly selected messages on each of these platforms.

On seeing the message, users could engage with the post by commenting, sharing, liking, and clicking on the link in the message, which took them to an educational website operational during the campaign period. The website provided more information about the risks of tobacco products, which was based on the government-sponsored health education campaigns. The details of the technology-enhanced implementation of the campaign and examples of messages with images for each platform are depicted in the related technical implementation paper from Reuter et al [15].

We captured responses to all campaigns messages posted during the study period. Approximately 35.68% (569/1595) of the campaign message posts received public comments (after excluding deleted comments or comments from user accounts blocked during the study period). This paper is focused on the analysis of the comments (n=1242) to the campaign. The scope of this research was observational with the intent to characterize the nature of public responses to an antismoking campaign. The intent was not to respond to the public comments, influence the comments, or further engage the audience.

### Data

Comments to the campaign messages were extracted from 3 social media platforms (Twitter, Facebook, and Instagram) using an automated tool described previously [15]. The research team collected comments both manually and automatically by logging into the comment moderation interfaces and analytics interfaces provided by each social media platform. Manual collection and automated collections were compared to ensure a complete overview of all responses was captured. The technical tool used in this study supports the following functions: (1) data import, (2) message generation based on randomization techniques, (3) message dissemination, (4) import and analysis of message comments, (5) collection and display of metrics related to message performance, and (6) reporting based on a predetermined data dictionary.

### Content Coding

We used a codebook defining coding categories to address the hypothesis. Two independent study team members coded the comments to the original posts, in terms of 7 coding categories: (1) tone of the comment (positive, negative, or neutral), (2) nature of contribution (toxic, healthy, or unclear/not applicable), (3) agreement with prevention message (agree, disagree, or seek clarification or advice), (4) mentions of government agency (yes or no), (5) policy/regulation (proregulation, antiregulation, neutral-regulation, or not applicable), (6) promotion/spam (yes or no), and (7) format (text only, meme/sticker/emoji/emoticon only, or both). Toxic contributions were defined as “a rude, disrespectful, or unreasonable comment that is likely to make other users leave a discussion” [27], whereas healthy contributions were defined as those using non-toxic language, those that were unclear or for which the classification was not applicable, or those using vague terms or emojis/stickers/emoticons, for which toxicity could not be determined.

As a first step, the coders coded 50 comments and discussed disagreements and results with the principal investigator to refine the initial codebook. Promotional and spam-like comments were identified as an emergent coding category.

To establish intercoder reliability, two coders independently coded 10% of the total sample (N=127). The overall agreement for the themes (94% agreement, κ=0.90) was substantial. The range of the coding agreement was acceptable and ranged from 92% to 100% (k=0.85 to 1). All disagreements were resolved by one of the investigators.

### Analysis

Chi-square analysis was used to test associations between the agreement with the message and tone of the response, and with the toxicity of the comments.

## Results

The final sample consisted of 1242 comments (Twitter: n=1004; Facebook: n=176; Instagram: n=62). Comments were predominantly text-based (1137/1242, 91.55%) and nonpromotional or nonspam posts (1222/1242, 98.39%). The highest proportion of comments were negative (531/1242, 42.75%), followed by neutral (354/1242, 28.50%), positive comments (126/1242, 10.14%), and other/unclear (231/1242, 18.60%). About 39.77% (494/1242) of the comments disagreed with the health messages, whereas 23.91% (297/1242) of the comments agreed or approved of the health messages. Most of the comments made healthy contributions (1048/1242, 84.38%), while about 12.88% (160/1242) of them were coded as toxic comments. Additionally, there were no mentions of government agencies in 99.44% (1235/1242) of the posts and only 6 of the comments that mentioned government agencies were antiregulation. Please refer to Table 1 for detailed results, including code categories and their corresponding definitions. Example comments were paraphrased to protect the identity of the individuals in this study.

**Table 1:** Sample characteristics of comments collected from Twitter, Facebook, and Instagram in response to a tobacco prevention campaign.

Category			Number of comments (N=1242), n (%)	Examples^a^
**Format**				
	Text only		1137 (91.55%)	“What do you mean by on average? What is the test subject? Mice?” “Kids may think that smoking makes them looks big with other people that smoke but it doesn't”
	Meme/sticker/emoji/emoticon only		36 (2.90%)	“ 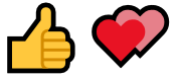 ” “ ;) ”
	Text and meme/sticker/emoji/emoticon		69 (5.56%)	“So are they saying that I should be rolling up a rat poison cigarette? Because if I must then I will 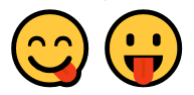 ” “Amen! :( ”
**Tone of the comment**				
	**Overall positive tone**		126 (10.14%)	
		Joyous		“Yeah I heard of that. If you smoke quit for your family and friends. Breathing fresh air is so much better than smoking!”
		Hopeful		“15 months and going strong after after 38 yrs.of smoking. Hope this continues!”
		Supportive		“Amen that's why I quit smoking”
		Unknown		“Nice. Cheers :)”
	**Overall negative tone**		531 (42.75%)	
		Anger		“You're stupid!”
		Fear		“That's a lot of people dying from smoking every year. Sooner or later we'll run out of people.”
		Sarcasm		“On average? What was your test subject – Mice?”
		Disgust		“Ugh Jeez. Smoking is very gross.”
		Sadness/despair		“Oh please don't say that--lost my husband when he was 39years old and we had 3 children. He had a horrific painful death”
		Unknown		“Smoking and drinking! Why don’t you get it morons..How could it possibly go wrong?”
	Overall neutral tone		354 (28.50%)	“Quit while you can” “No proof”
	Other/unclear		231 (18.60%)	“How many lives would be saved with no abortions?”
**Agreement **				
	Agreement (agrees with or approves of the message of the original post)		297 (23.91%)	“Yes, people like me now have COPD. I have never smoked just been around people who have” “Yeah! Sad should have been banned a long time ago!!!!”
	Disagreement (engages in disagreement/disbelief/criticism in response to the original post)		494 (39.77%)	“Nope, there's still polluted air btw” “Don't believe this propaganda, not based on facts !”
	Clarification and/or advice seeking (posts questions to seek more information or advice).		93 (7.49%)	“Again I ask...how do you recommend we quit quit smoking?” “& it's called 2nd hand smoke?”
	Other/unclear		358 (28.82%)	“This is how I will look tomorrow.” “Also with the farm”
**Nature of contribution**				
	Toxic contribution (a rude, disrespectful, or unreasonable comment that is likely to make other users leave a discussion)		160 (12.88%)	“Here’s what I have to say: Haters gonna hate,bitches gonna bitch.” “FUCK YOU”
	Healthy contribution (comments that did not contain toxic words/language)		1048 (84.38%)	“All smokers read this for sure” “Unfortunately for some, they are so addicted that they were happier than if they had quit . This is een though they would have lived longer, they would have been miserable.”
	Unclear/not applicable (comments with vague terms or emojis/stickers/emoticons, for which toxicity could not be determined)		34 (2.74%)	“That reminds me of this (picture of cigarette with smoke attached)”
**Mentions of government agency **				
	Yes (direct or implied mentions of the government and/or government agencies such as FDA^b^/CDC^c^/NIH^d^ in the comment)		7 (0.56%)	“How can this be allowed....It's called the FDA” “The FDA stands for ‘FOR DEATH AWAITS,’,”
	No (no direct or implied mention of the government and/or government agencies such as FDA/CDC/NIH in the comment)		1235 (99.44%)	“Look, we hate smoking, and am all in favor of the heavy hand of government, but your tweet is a flat out lie. Why not” “#JustBeHonest about it!” “I have a right given to me by our great Bill of rights, to smoke. ,Pre-manufactured foods and the nasty water cause more deaths.”
**Policy/regulation^e^**				
	Proregulation (comment references regulation[s] to show support)		1 (0.08%)	“I support this am all in favor of the heavy hand of antismoking government policies”
	Antiregulation (comment references regulation[s] to make an antiregulatory statement)		6 (0.48%)	“The FDA puts these things in cigarettes …They are putting the same poisons in your food and water. But let's just keep blaming tobacco product's. Wake up peoplecancer is a man-made disease It is amazing how the government makes something like a weed illegal a weed that has been here long before any of us.... just amazing” “If no one ate, maybe the cancer rate would go down! If nobody drank, maybe the drunk driving deaths would go down! Maybe you should mind your own business!Just maybe taking the chemicals out of the tobacco that the government (fda) says is okay, maybe cancer would go down!”
	Not applicable to regulation (the comment was not about regulation and/or did not reference regulatory bodies to make a pro-or antiregulatory stance)		1235 (99.44%)	“There are nasty things in cigs”
**Promotion/spam **				
	Yes (Comments that promoted either the user's social media or others account, products, or services)		20 (1.61%)	“Follow me fams :)”
	No		1222 (98.39%)	N/A^f^

^a^Example comments are paraphrased to protect user privacy.

^b^FDA: Food and Drug Administration.

^c^CDC: Centers for Diseases Control and Prevention.

^d^NIH: National Institutes of Health.

^e^Pro- or antiregulation categories were applicable only when the response mentioned a government agency in the comment.

^f^N/A: Not applicable.

Agreement with the message was significantly associated with the tone of the response (χ2 =1000, *df*=9; *P*=0.01). Most of the comments that employed a positive tone predominantly agreed with health messages (84.13%, 106/126; eg, “Amen! I agree with this!”), and few expressed disagreement (2.38%, 3/128; eg, “I am not sure I agree but I am willing to listen”) or asked for clarifications (0.79%, 1/128; eg, “Would it be possible to explain how that would work?”). Comments that employed a negative tone mostly disagreed with prevention messages (69.11%, 367/531; eg, “Stop taking us for a ride!”), followed by those that asked for clarifications (11.11%, 59/531; eg, “Why do you let these companies put all the chemicals in everything?”) or expressed agreement (6.78%, 36/531; eg, “Smoking is indeed a filthy habit”). The comments that used a neutral tone mostly expressed agreement (42.94%, 152/354; eg, “Yes, smoking kills,” followed by those that expressed disagreement (34.75%, 123/354; eg, “This gives me a better reason to smoke”) or sought clarifications (6.21%, 22/354; eg, “How would that happen?”).

Agreement with the message was also significantly associated with the toxicity of the comments (χ2 =176.23, *df*=6; *P*=0.01). The majority of the toxic comments disagreed with prevention messages (71.25%, 114/160; eg, “Bullshit! You have no data to support that!.”) whereas few agreed with the prevention messages (2.50%, 4/160; eg, “Smoking shit will kill you”) or sought clarifications (12.50%, 20/160; eg, “What the fuck am I looking at?”). Among those comments that made healthy contributions, about one-third of the comments disagreed with the messages (36.26%, 380/1048; eg, “Air pollution causes more deaths than smoking”) followed by ones that agreed with the messages (27.86%, 292/1048; eg, “Amen, that’s why I quit”) or sought clarifications (6.97%, 73/1048; eg, “Any ideas on how to end this epidemic?”).

## Discussion

The findings support our hypothesis. Exposure to tobacco prevention health messages on social media stimulated primarily healthy rather than toxic contributions. However, negative and toxic comments mostly disagreed with the health messages. The use of toxic language constitutes incivility online, which is also shown to exacerbate polarity of opinions on social media [28], thus generating more incivility [29]. In general, incivility in online discourse is predominant in social media involving the communication of scientific data or findings [30]. Prevalence of such incivility counteracts public health efforts to educate and inform the public about scientifically proven health risks of tobacco use.

Managing toxic comments on social media is a crucial issue to be addressed for successful health campaigns. Previous research also suggests that public health campaigns on social media may make public health groups a target for counter campaigns with a large volume of anticampaign posts questioning the intent or scientific basis of the messages [11,12]. As such, it is crucial to incorporate comment moderation protocols into tobacco prevention campaigns on social media. This could include automated moderation tools that detect toxic comments to support fast response and moderation [26,31-34], educating the public about conversational techniques that sustain the productivity of online discourses, or deletion of toxic comments and suspension of associated accounts.

There is a need to address symptoms of polarity on social media such as language toxicity or use of negative tone in future health campaigns in the form of counterargumentation or effective moderation of these discussions. Current community standards developed by social media platforms attempt to address this issue by defining benchmarks and policies for online discourse [35,36]. For this health campaign experiment, we blocked 26 users (2.1%; 26 on Facebook, 0 on Twitter, and 0 on Instagram) and deleted 2 of the 1242 comments (0.16%; 2 on Facebook, 0 on Twitter, and 0 on Instagram) that used toxic or particularly offensive language (eg, “Get that stick out of your ass,” “Babies don’t smoke but they may have cancer too,” and “Stop bombarding Facebook with the no smoking bullshit”). Future work may consider a larger sample of posts from blocked users in terms of the tone, agreement with campaign messages, and nature of contribution. To safeguard free speech online, a moderator could respond to comments that use toxic language and a negative tone using countermessaging strategies to engage those individuals in further dialogue. Emerging evidence suggests that styles of interactive moderation, where moderators request individuals using uncivil or toxic language to use more civil language, differ in their effectiveness [37]. Ziegele et al [37] found that social style of moderation (eg, moderation involving complementing comments informally or creating an informal and pleasant discussion) was associated with decreased incivility. Regulative style of moderation (eg, regulation involving checking for facts complaining about comments, requesting more civil behavior, or pointing to violations of rules) was linked to increased incivility [37]. More work at the intersection of moderation style and counter speech language strategy is needed to address increasing toxicity online. Online health-related campaigns can also benefit from automated detection of incivility, including use of toxic language. Recent efforts to leverage artificial intelligence to identify and address specific behaviors can also play a crucial role in managing the undesirable effects of uncivil discourse [38]. Future interventions can also develop automated alerts for toxic language to inform a moderator that a comment should be looked at.

The reactance theory offers a useful framework to contextualize findings. Most of the negative or toxic comments disagreed with the prevention messages. Toxicity and negative tone, as such, appear to be symptoms of reactance to health messages. Evidence also suggests that reactance to persuasion messages can also lead to source degradation, which is defined as the use of aggression or hostility toward a threatening agent [39]. Thus, minimizing audience reactance to a message is key. Tactics to do this include avoiding overtly freedom-threatening language (eg, telling people they must stop smoking) [40], emphasizing the audience’s freedom to choose [40], avoiding attacks to one’s identity as a smoker [19], and complementing fear-producing messages with an efficacy boosting message (eg, if a message discusses connections between smoking and lung cancer, it should also provide concrete ways for users to stop smoking) [38]. Additionally, because toxic online comments may bias other users against the health message or message source, campaigns can take steps to prevent this from happening. Inoculation theory provides one pathway to accomplish this. This approach involves “inoculating” the audience against possible counterclaims or opposing messages, (eg, warning an audience that “Some argue that vaccines cause autism but science has proven that this is not true”). Allowing the audience to be aware of potential counterattacks to the message can help them better resist said counterattacks and can potentially address reactance and improve health outcomes [41]. Future research can investigate associations between the audience response and inoculation strategies for tobacco prevention campaigns. Developing methods to assess reactivity to health message exposure on social media platforms and examining the nature of online engagement with health messages also offers valuable directions for future work.

Our findings should be viewed in light of several limitations. First, results pertain to public reactions to a specific health-related context of smoking prevention. Future studies may use the automated tool used in this study to examine reactions in other health contexts. Our data is also limited to three social media platforms (Twitter, Instagram, and Facebook) and may not be generalizable to other platforms such as Reddit, YouTube, or SnapChat. Blocking users associated with toxic comments during the campaign may have potentially biased the contribution and tone of the subsequent comments. Future studies may consider examining the effects of toxic comments on subsequent discussions in online health campaigns. In this study, two members of the research team undertook content moderation decisions. We were unable to measure the effectiveness of different moderating strategies such as blocking versus hiding toxic comments. Excluding blocked comments, potentially predominantly toxic, from the analytic sample may have influenced the proportion of toxic comments in our final sample. Moderators also did not respond to any public comments, which may or may not have impacted the trajectory and quality of conversations. Another limitation of this study pertained to treating each comment, including replies to other users’ comments (73/1242, 5.9% of the analytic sample), as an independent unit of observation. While the sample of replies to other users’ comments was small, it may have influenced the proportion of coding categories to some extent. We were unable to test associations between some of the categories (eg, ”mentions of government agency” and ”agreement;” “mentions of government agency” and “toxicity”) due to low sample sizes. We were also unable to characterize blocked user comments in terms of their agreement with the campaign message, tone, and nature of contribution due to low sample sizes.

Our research offers insights about the nature of public response to tobacco prevention messages. While revealing concerning trends of toxicity and use of negative tone while expressing disagreements with the prevention messages, our findings also encourage the use of social media to promote dialogue about controversial health topics such as smoking. Future health interventions should develop methods including technology-enhanced techniques to manage toxic user comments and to educate social media users about the harmful effects of toxicity and negative tone in the overall discourse about public health issues.

## Appendix

Multimedia Appendix 1 List of parameterized message templates used in the study.

## Abbreviations

FDA: Food and Drug Administration
NIH: National Institutes of Health
